# Health-Related Quality of Life in Chinese Patients with Mild and Moderately Active Ulcerative Colitis

**DOI:** 10.1371/journal.pone.0124211

**Published:** 2015-04-27

**Authors:** Kai Zheng, Shengsheng Zhang, Chuijie Wang, Wenxia Zhao, Hong Shen

**Affiliations:** 1 Department of Gastroenterology, Affiliated Hospital of Nanjing University of Chinese Medicine, Nanjing, Jiangsu, China; 2 Department of Gastroenterology, Beijing Hospital of Chinese Medicine, Beijing, China; 3 Department of Gastroenterology, Affiliated Hospital of Liaoning University of Chinese Medicine, Shenyang, Liaoning, China; 4 Department of Gastroenterology, the First Affiliated Hospital of Henan University of Chinese Medicine, Zhengzhou, Henan, China; University Hospital Llandough, UNITED KINGDOM

## Abstract

**Background:**

Ulcerative colitis (UC) impairs the health-related quality of life (HRQOL). The difference in HRQOL between patients with mild and moderately active UC is not well-defined. Few studies have been conducted to explore the factors that influence HRQOL in Chinese patients. Our study aims were to (1) compare HRQOL of mildly active UC patients with moderate patients; (2) explore the factors that influence HRQOL in Chinese patients with UC; and (3) analyze demographic and disease characteristics of UC in China.

**Methods:**

A total of 110 mild and 114 moderate patients with UC were enrolled. The demographic and disease characteristics were recorded. HRQOL was measured by the Chinese version of the inflammatory bowel disease questionnaire (IBDQ) between mild and moderate patients, male and female patients, and different disease distributions. Stepwise regression analysis was used to assess factors influencing the IBDQ score.

**Results:**

Patients with moderate UC had significantly lower IBDQ total scores compared to patients with mild UC (P=0.001). The IBDQ total score had a negative correlation with the Mayo score (r=–0.263, P<0.001). Stepwise regression analysis showed that the disease activity index and gender had an influence on the IBDQ total score (P<0.05). The female patients had a lower score than the male patients (P<0.05), especially in the emotional function domain (P=0.002). Different disease distributions were not statistically significant in the IBDQ total score (P=0.183).

**Conclusions:**

UC has a negative influence on HRQOL. HRQOL in patients with moderate UC was lower than HRQOL in patients with mild UC, as measured by the IBDQ. UC disease activity has a negative correlation with HRQOL. Gender and the disease activity index are important factors involved in the impairment of HRQOL in Chinese patients with UC. Chinese females may benefit from increased psychological care as part of UC therapy.

## Introduction

Ulcerative colitis (UC), a form of inflammatory bowel disease (IBD), is an idiopathic, chronic inflammatory disorder of the colonic mucosa. The main symptoms of UC include diarrhea, rectal bleeding, and abdominal pain. Systemic features, such as fever, fatigue, and weight loss are more common if all or most of the colon is involved in patients with UC. Most commonly, UC follows a chronic intermittent course with periods of remission interspersed with relapse lasting weeks to months. Patients with UC have been reported to experience an exacerbation of symptoms prior to remission [1,2]. The disease profoundly impacts patients’ quality of life [3,4].

The concept of health-related quality of life (HRQOL) encompasses a more complete evaluation of the effects of the disease, incorporating physical, emotional, and social aspects of health perception and health functioning [4]. Measurement of HRQOL is becoming more prominent in studies of patients with IBD and evaluation of disease [5,6]. Instruments that measure the HRQOL can be divided into generic instruments or disease-specific questionnaires. One such disease-specific instrument, the inflammatory bowel disease questionnaire (IBDQ), as developed by Guyatt and Irvine [7,8], has been shown to be valuable in assessing patients with IBD [9–11] and has widely been used in assessing the therapeutic efficacy in clinical trials [5,6,12,13].

The HRQOL correlates with disease activity in patients with UC [14–16]. IBDQ scores are significantly different between patients with active disease and patients in remission [9,17], as well as patients with mild and moderate-severe disease [11,18]; however, no studies have been conducted involving the HRQOL in patients with mild and moderate UC.

Some factors have a significant effect on the HRQOL in patients with UC. Disease characteristics, such as disease and endoscopic activity indices, correlate with the HRQOL, but disease distribution, duration of disease, and hospital admission, as well as demographic parameters, such as educational status, economic status, and marital status, do not significantly affect the IBDQ total scores [15,19]; factors, including gender and age, are controversial. Some studies support the effect of gender and age related to the HRQOL [20,21], while others do not [15,19]. In China, a study explored factors affecting 92 patients with UC and Crohn disease in one province [22], but no large-sample study involving the factors which influence the HRQOL in patients with UC in different regions of China has been conducted.

The aims of our study were to (1) compare HRQOL of mildly active UC patients with moderate patients; (2) explore the factors that influence the HRQOL in Chinese patients with UC; and (3) analyze demographic and disease characteristics of UC in China.

## Methods

### Study population

Patients with mild and moderately active UC were recruited from outpatients and inpatients at the Affiliated Hospital of Nanjing University of Chinese Medicine, Beijing Hospital of Chinese Medicine, Affiliated Hospital of Liaoning University of Chinese Medicine, and the First Affiliated Hospital of Henan University of Chinese Medicine in this cross-sectional study. The inclusion criteria were as follows: participants were between 18 and 65 years of age and had a confirmed diagnosis of mild or moderate UC. Before enrollment, all of the patients underwent a colonoscopy examination within 1 month. The diagnosis of UC was made according to the UC practice guidelines in adults (update) [1]. The Mayo score was used to assess disease activity (Table 1) [23]; specifically, mild UC was defined as a Mayo score of 3–5 points, moderate UC was defined as a Mayo score of 6–10 points, and remission was defined as a Mayo score ≤ 2 [2,24]. The exclusion criteria were the inability to comprehend or complete the IBDQ or the presence of co-existing diseases, including congestive heart failure, a history of a cerebrovascular accident, renal insufficiency, cirrhosis, peptic ulcers, diabetes mellitus, malignancies, and psychiatric disorders impacting HRQOL.

**Table 1:** Mayo score system.

Parameters	Subscore, 0–3
Stool frequency	0 = Normal number of stools for this patient
	1 = 1–2 stools more than normal
	2 = 3–4 stools more than normal
	3 = 5 or more stools more than normal
Rectal bleeding	0 = No blood seen
	1 = Streaks of blood with stool less than one-half of the time
	2 = Obvious blood with stool most of the time
	3 = Blood alone passes
Findings on endoscopy	0 = Normal or inactive disease
	1 = Mild disease (erythema, decreased vascular pattern, and mild friability)
	2 = Moderate disease (marked erythema, lack of vascular pattern, friability, and erosions)
	3 = Severe disease (spontaneous bleeding and ulcerations)
Physician’s global assessment	0 = Normal
	1 = Mild disease
	2 = Moderate disease
	3 = Severe disease

Two hundred forty-five Chinese patients with confirmed mild or moderate UC were enrolled. Twenty-one patients were excluded because of co-existing diseases which may impact the HRQOL. Finally, 110 mild and 114 moderate patients with UC were included.

The demographic parameters recorded were ID number, gender, and age. The disease characteristics included disease distribution, endoscopic grade, Mayo score, extra-intestinal manifestations, duration of disease, hospital admission, laboratory testing results, and medications.

### Instruments

The IBDQ is a disease-specific questionnaire which contains 32 items categorized under four domains: bowel symptoms (10 questions); systemic symptoms (5 questions); emotional function (12 questions); and social function (5 questions). Responses are scored on a 7-point Likert scale in which 7 corresponds to the highest level of function. The final score ranges from 32 to 224; the higher the score, the better the HRQOL.

We obtained the questionnaire license agreement: use of the IBDQ, authored by Dr. Jan Irvine, was made under license from McMaster University, Hamilton, Canada. Dr. Jan Irvine authorised the Chinese version of the IBDQ, which is a translation from the original English version. Patients were requested to complete the Chinese IBDQ to reflect the HRQOL of the previous 2 weeks.

### Statistics

Statistical analysis was performed with SPSS (version 17.0 for Windows, SPSS Inc., Chicago, IL, USA). Frequency, median, and mean ± standard deviation (SD) were used for descriptive statistics. Student’s t-test and analysis of variance were used for continuous variables with a normal distribution. The Mann–Whitney U-test and Kruskal–Wallis test were used for continuous variables with abnormal distribution. A chi-square test was used for categorical variables. Linear regression was used to assess the association and Spearman correlation coefficients were used to assess the correlation between the IBDQ score and Mayo score. Stepwise regression analysis was used to assess factors influencing the IBDQ score. The IBDQ total scores were used as dependent variables, and gender, age, hospital admission, Mayo score, extra-intestinal manifestations, disease duration, endoscopic grade, and disease distribution were used as explanatory variables. The choice of explanatory variables was based on literature reviews [15,16,20,25–27] and clinical experience. Three explanatory variables (disease duration, endoscopic grade, and disease distribution) were categorical variables with three levels. These variables were transformed appropriately to three sets of dummy variables. For all analyses, a P value <0.05 was considered statistically significant.

### Ethics

The study was approved by the Institutional Review Board of Human Research of the Affiliated Hospital of Nanjing University of Chinese Medicine. The patients gave their informed consent before enrollment. Participants provided their written informed consent to participate in this study.

## Results

### Patient characteristics

The demographic and disease characteristics of all 110 mild and 114 moderately active patients are listed in Table 2. There were no significant differences in age, gender, extra-intestinal manifestations, disease duration, and hospital admission between the patients with mild and moderate UC. The disease distribution and endoscopic grade were statistically significant between the mild and moderate patients (P<0.05); specifically, 45.5% and 14.5% of the patients with mild UC had proctitis and extensive colitis compared to 34.2% and 28.1% in the patients with moderate UC, respectively. Moderate and severe findings were noted on endoscopy in 57.9% and 34.2% of patients with moderate UC compared to 28.2% and 29.1% in patients with mild UC, respectively.

**Table 2:** Demographic and disease characteristics of the patients.

Parameters	All patients (n = 224)	Mild (n = 110)	Moderate (n = 114)	P value
Demographics				
Age (years)				0.11
Median (IQR)	38.00(29.00–47.00)	37.00(38.00–45.00)	39.00(32.00–49.00)	
Range	19–65	19–63	19–65	
Gender, n (%)				0.39
Male	130(58.0%)	67(60.9%)	63(55.3%)	
Female	94(42.0%)	43(39.1%)	51(44.7%)	
Disease characteristics				
Disease distribution, n (%)				0.04
Proctitis	89(39.7%)	50(45.5%)	39(34.2%)	
Left-sided	87(38.8%)	44(40.0%)	43(37.7%)	
Extensive	48(21.5%)	16(14.5%)	32(28.1%)	
Endoscopic grade, n (%)				0.00
Mild findings on endoscopy	56(25.0%)	47(42.7%)	9(7.9%)	
Moderate findings on endoscopy	97(43.3%)	31(28.2%)	66(57.9%)	
Severe findings on endoscopy	71(31.7%)	32(29.1%)	39(34.2%)	
Extra-intestinal manifestations, n (%)	3(1.3%)	1(0.9%)	2(1.8%)	1.00
Disease duration				0.98
<1 year	36(16.1%)	18(16.4%)	18(15.8%)	
1–5 years	140(62.5%)	68(61.8%)	72(63.1%)	
>5 years	48(21.4%)	24(21.8%)	24(21.1%)	
Range (years)	0.5–30	0.5–30	0.5–20	
Inpatient or outpatient, n (%)				0.44
Hospitalized	21(9.4%)	12(10.9%)	9(7.9%)	
Outpatient	203(90.6%)	98(89.1%)	105(92.1%)	
Laboratory examination				
Leukocytes (10^9^/L)	6.24±1.85	5.78±1.55	6.68±2.02	0.00
Hemoglobin (g/L)	134.03±20.24	135.32±19.74	132.78±20.71	0.35
CRP (mg/L)	5.26±5.09^a^	4.34±3.88^b^	6.10±5.89^c^	0.03
Medications, n (%)				
5-aminosalicylates	89	53	36	
5-aminosalicylates+corticosteroids	32	5	27	
5-aminosalicylates+azathioprine	5	0	5	
5-aminosalicylates+Chinese herbs	75	32	43	
Chinese herbs	23	20	3	

^a^n = 148

^b^n = 71

^c^n = 77

The patients’ ages were non-normally distributed (Table 2), and ranged from 19 to 65 years. The median age of patients was 38 years. The frequency of 28–30 years was higher than the other ages. There were more males than females included in UC patients (58% vs. 42%). Only 1.3% of patients had extra-intestinal manifestations, including axial arthropathy and erythema nodosum. Of the patients, 90.6% were outpatients. The leukocyte counts and CRP levels in patients with mild UC were significantly lower than the leukocyte counts and CRP levels in the patients with moderate UC (P<0.05; Table 2); however, there was no significant difference between the two groups with respect to the hemoglobin levels. The patients’ medications are listed in Table 2.

### HRQOL of mild and moderately active UC

The mean ± SD IBDQ total scores of patients with mild and moderate UC were 156.20±24.99 and 143.94±28.10, respectively (Table 3); the patients with moderate UC had a significantly lower total score compared with the patients with mild UC (P = 0.001). The moderate patients’ mean or median of four domains was also significantly lower than the patients with mild UC (P<0.05), especially with respect to the bowel symptoms domain (P = 0.001).

**Table 3:** IBDQ total and domain score in patients with mild and moderate UC.

Item	Mild (n = 110)	Moderate (n = 114)	P Value
Bowel symptoms	48.82±8.37	44.84±8.96	0.001
Systemic symptoms	22.47±4.61	21.13±5.20	0.042
Emotional function	58.84±9.94	54.36±11.50	0.002
Social function	27.00(23.75–30.00)	24.00(18.00–30.00)	0.011
Total score	156.20±24.99	143.94±28.10	0.001

### Correlation between HRQOL and disease activity

The mean IBDQ total score of patients with mild and moderately active UC was 149.76±27.58. The Spearman correlation coefficients between the IBDQ domain score and disease activity index are shown in Table 4. The Mayo score includes stool frequency, rectal bleeding, findings on endoscopy, and the physician’s global assessment. Bowel symptoms are one component of the Mayo score. The correlation with Mayo scores appeared to be weaker with respect to systemic symptoms, emotional function, and social function domain scores than the bowel symptoms; social function had the weakest correlation (r2 = –0.026; P<0.05). A negative correlation existed between the IBDQ total and Mayo scores (r2 = –0.069, P<0.001), Although the correlation was weak because many factors, such as disease activity index, gender, education level, employment status, a family history of IBD, smoking status, and previous surgical resection, may influence HRQOL. S1 Fig represents the relationship between the IBDQ total and Mayo scores by linear regression (y = –4.144x+175.585, P<0.001).

**Table 4:** Correlation between IBDQ total and domain scores and Mayo score.

Item	Mayo score		
	r	r^2^	P Value
Bowel symptoms	-0.326	-0.106	0.000
Systemic symptoms	-0.210	-0.044	0.002
Emotional function	-0.202	-0.041	0.002
Social function	-0.160	-0.026	0.017
Total score	-0.263	-0.069	0.000

### Factors influence the HRQOL

Linear regression analysis showed that higher disease activity index and female gender were associated with a lower total IBDQ score (both P<0.05), even after adjustment for age, disease distribution, endoscopic grade, extra-intestinal manifestations, disease duration, and hospital admission (Table 5).

**Table 5:** Factors influencing IBDQ total score in patients with UC.

Variables	β	t	P Value
IBDQ total score			
Mayo score	-0.213	-2.535	0.012
Gender	-0.193	-2.953	0.003
Age	-0.65	-0.994	0.322
Hospital admission	-0.33	-0.512	0.609
Extra-intestinal manifestations	-0.76	-1.160	0.247
Disease duration			
1–5 years vs. <1 year	-0.017	-0.188	0.851
>5 years vs. <1 year	-0.003	-0.039	0.969
Endoscopic grade			
Moderate vs. abnormal	0.020	0.256	0.798
Severe vs. abnormal	-0.074	-0.840	0.402
Disease distribution			
Left-sided vs. proctitis	-0.031	-0.434	0.665
Extensive vs. proctitis	0.079	1.103	0.271
	Adjusted R^2^ 0.092		

### HRQOL of male and female patients

Altogether, 130 male and 94 female patients with UC were enrolled. The mean ± SD IBDQ total score of female patients was 142.91±29.45, which was significantly lower than male patients (158.37±24.32, P<0.01). The mean or median of bowel symptoms, systemic symptoms, emotional function, and social function was lower in female patients than male patients (Table 6). Four domains between male and female patients were statistically significant (P<0.05), especially the emotional function domain (P = 0.002).

**Table 6:** IBDQ total and domain score in male and female patients.

Item	Male (n = 130)	Female (n = 94)	P Value
Bowel symptoms	47.95±8.63	45.19±9.03	0.021
Systemic symptoms	22.45±4.74	20.88±5.12	0.019
Emotional function	59.50(52.00–65.00)	54.00(45.00–63.00)	0.002
Social function	27.00(21.75–30.00)	24.00(18.00–29.00)	0.018
Total score	158.37±24.32	142.91±29.45	0.002

### HRQOL of different disease distribution

Eighty-nine, 87, and 48 patients with UC had proctitis, left-sided colitis, and extensive colitis, respectively. The median IBDQ total scores for patients with proctitis, left-sided colitis, and extensive colitis were 152.00, 154.00, and 160.50, respectively (Table 7). The differences in disease distribution were not statistically significant with respect to the IBDQ total score (P = 0.183) and four domains (P>0.05).

**Table 7:** IBDQ total and domain score in different disease distributions.

Item	Proctitis (n = 89)	Left-side (n = 87)	Extensive (n = 48)	P Value
Bowel symptoms	46.76±8.39	47.02±9.18	46.44±9.38	0.935
Systemic symptoms	21.93±4.67	22.26±5.18	20.67±4.97	0.188
Emotional function	56.06±10.57	57.61±11.74	54.65±11.59	0.326
Social function	26.00(21.00–29.50)	26.00(21.00–30.00)	25.50(16.00–30.00)	0.598
Total score	152.00(129.00–166.50)	154.00(138.00–169.00)	160.50(132.50–177.50)	0.183

### HRQOL of corticosteroids

Thirty-six and 27 patients with moderate UC received 5-aminosalicylates and 5-aminosalicylates and corticosteroids, respectively. Patients receiving 5-aminosalicylates and corticosteroids had lower IBDQ scores than patients only receiving 5-aminosalicylates (Table 8). The mean of the total score, bowel symptoms, systemic symptoms, and emotional function symptoms was significantly lower in patients taking 5-aminosalicylates and corticosteroids (P<0.05). The difference in social function was not statistically significant (P>0.05).

**Table 8:** IBDQ total and domain score in patients with moderate UC and different medications.

Item	5-aminosalicylates (n = 36)	5-aminosalicylates+corticosteroids (n = 27)	P Value
Bowel symptoms	46.92±6.85	40.33±5.29	0.000
Systemic symptoms	21.67±4.21	19.52±3.12	0.029
Emotional function	55.75±8.46	50.26±6.31	0.005
Social function	24.22±6.14	22.04±5.96	0.162
Total score	148.56±20.81	132.15±15.38	0.001

## Discussion

The incidence of UC in China is less than Europe and North America, but is increasing rapidly [28]. An increasing number of Chinese patients with UC have a low HRQOL [22]. Zhou [22] studied 92 patients with UC and Crohn’s disease in Zhejiang, China. HRQOL was impaired in patients with UC and Crohn’s disease. Only the disease activity index and employment status affected HRQOL in the Zhou study [22]. Gender had no impact on the HRQOL. We described the demographic and disease characteristics and HRQOL features of patients with UC in China. We analyzed 224 patients with UC in 4 provinces of China and showed that gender and the disease activity index are important factors involved in impairment of the HRQOL. Of note, the female patients had a lower HRQOL. Gender appeared to be most strongly related to IBDQ total scores in the current study and other studies [20,25]; however, the influence of gender on HRQOL remains controversial and gender has not been reported to be a significant predictor of HRQOL in some studies [14,29]. In the current study, female patients had lower IBDQ total scores than males, especially with respect to emotional function compared to other domains. Chinese female patients with UC had more emotional problems compared to male patients [30]. This may be due to the traditional Oriental introversion of Chinese females that makes the results different from other studies in other countries. Psychological disorders in patients with IBD contribute to a poor HRQOL. Treatment of psychological disorders in patients with IBD disease has the potential to improve the HRQOL [26], thus implying that Chinese females need more psychological care in conjunction with UC therapy.

Patients with severe UC require hospitalization. Severe UC seriously affects the HRQOL with respect to bowel symptoms, systemic symptoms, emotional function, and social function domains. Nevertheless, patients with mild and moderate UC are mostly outpatients. The mean values of the IBDQ total score in patients with mild and moderate UC were <170 in the current study, while patients in remission have a score ≥170 [5,8]. The HRQOL in patients with mild and moderate UC was affected. The mean or median values of the IBDQ total score and four domain scores in patients with moderate UC were also lower than the HRQOL in patients with mild UC, especially with respect to the bowel symptoms domain. Thus, there was a difference in HRQOL between patients with mild and moderate UC. Our study showed the HRQOL to have a negative correlation with Mayo scores. Patients receiving corticosteroids and 5-aminosalicylates had an impaired HRQOL compared to patients who only received 5-aminosalicylates in the current study. Patients receiving corticosteroids had a worse HRQOL, which may be due to complications and adverse reactions to corticosteroids [31].

The age of patients with active UC in our study did not have a normal distribution. We suggest the chief reason for the non-normal distribution was due to age of UC onset. UC has a bimodal pattern of incidence, with the focal onset peak between ages 20 and 30 years, especially in the 30 years, and a second smaller peak between ages 60 and 70 years [32]. The peak of age distribution in our study was at 28–30 years; however, we did not identify the second smaller peak in Chinese patients. The second reason for the non-normal distribution was we did not include patients <18 years of age in our study. In most studies involving UC, there is a slight male predominance [32]. Zhou [22] analyzed 52 patients with UC in Zhejiang province, China; the male-to-female ratio was 1.26. Jiang [28] analyzed 10,218 patients with UC in China; the male-to-female ratio was 1.09. We analyzed 224 patients with UC in four provinces of China; there still tended to be a slight male predominance, with a male-to-female ratio of 1.38. Of the patients in the current study, 1.3% had extra-intestinal manifestations, which is less than the report (6.1%) by Jiang [28] in China. Age, extra-intestinal manifestations, and hospital admission did not correlate with the HRQOL in our study.

Leukocyte counts and CRP levels in patients with mild UC were lower than the leukocyte counts and CRP levels in patients with moderate UC, implying that disease progressed with inflammatory infiltration. The disease distribution and endoscopic grades between patients with mild and moderate UC were significantly different in the current study, implying that the disease progressed with an expanded disease distribution and more severe endoscopic manifestations. The disease activity correlated with disease distribution and endoscopic grade; however, disease distribution and endoscopic grade did not the predictors of HRQOL in our study. HRQOL included bowel symptoms, systemic symptoms, emotional function, and social function. Disease distribution and endoscopic grade were connected with bowel symptoms, but not decisive factors regarding emotional and social functions. Thus, the correlations were weaker between HRQOL, and disease distribution and endoscopic grade. Based on stepwise regression analysis after controlling for disease activity, disease distribution and endoscopic grade appeared to be disease activity-related, and therefore there was a diminishing correlation with the HRQOL. Different disease distributions were not statistically significant with respect to the IBDQ score. The disease activity index (Mayo score) had a negative correlation with the HRQOL in the current study, which is in agreement with previous reports [15,16].

There were several limitations to our study. We did not recruit patients in remission to compare with patients who had mild UC. Education level, employment status, a family history of IBD, smoking status, and previous surgical resection are some factors that we could further explore with respect to HRQOL in patients with UC. The relationship between fecal calprotectin levels and disease activity index will be determined in corollary studies.

## Conclusion

The HRQOL in patients with moderately active UC was less than the HRQOL in patients with mild UC. The HRQOL has a negative correlation with UC disease activity. IBDQ is a valid and reliable assessment tool that reflects the HRQOL of patients with UC. Factors, such as gender and the disease activity index (Mayo score), correlate with the HRQOL in China, but age, disease distribution, endoscopic grade, extra-intestinal manifestations, disease duration, and hospital admission have no correlations. Chinese females may benefit from additional psychological care in UC therapy.

## Supporting Information

S1 Dataset Research data. The research data used in this study.
(XLS)

S1 Fig Relationship between IBDQ total and Mayo scores. The disease activity index (Mayo score) had a negative correlation with the HRQOL.
(TIFF)

S1 File Chinese version of the IBDQ. (PDF)
