# Supplementary figures and images for: Health-Related Quality of Life in Chinese Patients with Mild and Moderately Active Ulcerative Colitis
Source: PLoS One. 2015 Apr 27;10(4):e0124211. doi: 10.1371/journal.pone.0124211 (PMC4411120; doi:10.1371/journal.pone.0124211)

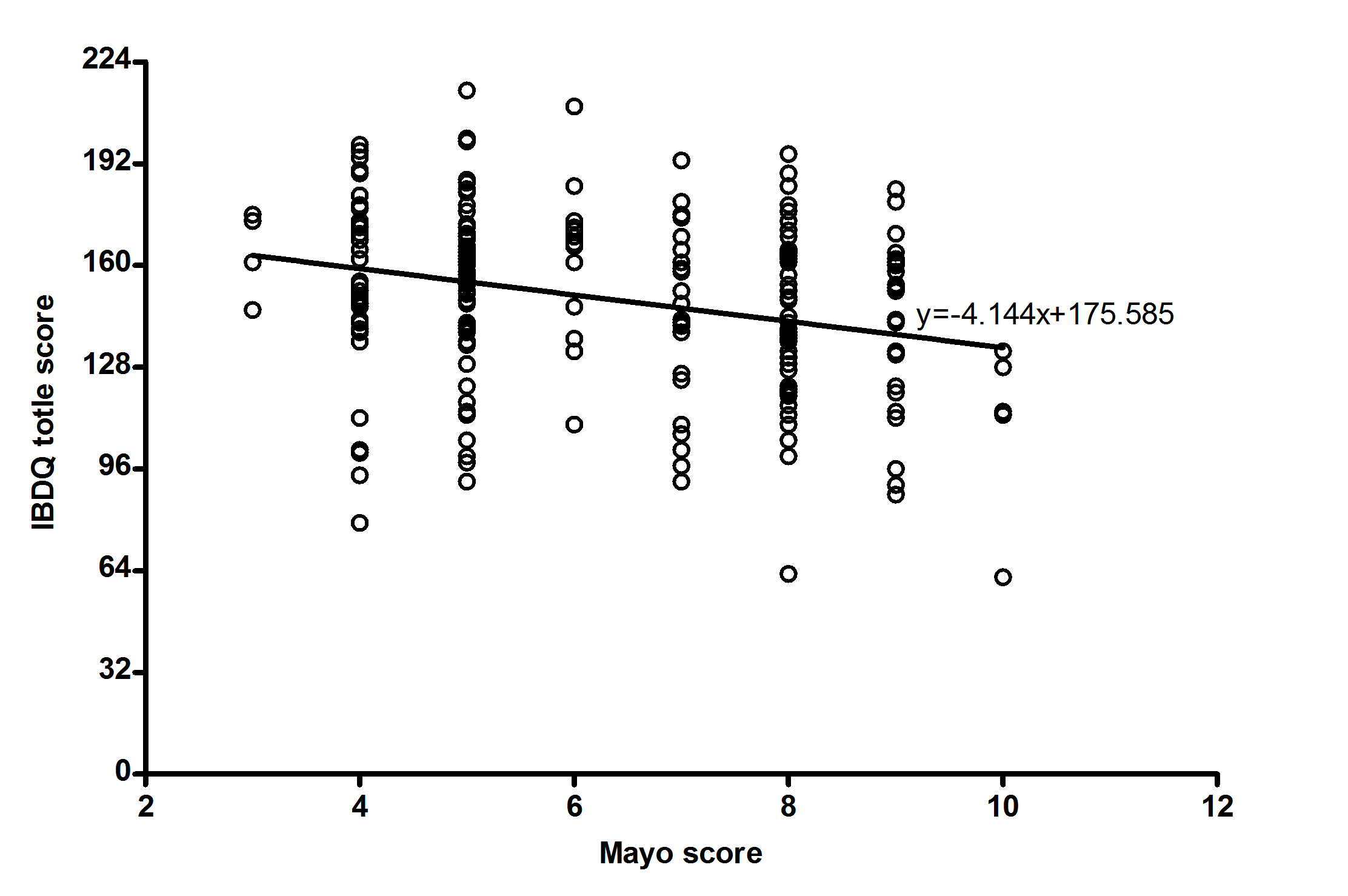

Supplement: S1 Fig — The disease activity index (Mayo score) had a negative correlation with the HRQOL. (TIFF) [file pone.0124211.s002.tiff]
