# Supplementary material for: Health-Related Quality of Life in Chinese Patients with Mild and Moderately Active Ulcerative Colitis
Source: PLoS One. 2015 Apr 27;10(4):e0124211. doi: 10.1371/journal.pone.0124211 (PMC4411120; doi:10.1371/journal.pone.0124211)
Supplement: S1 File — (PDF) [file pone.0124211.s003.pdf]

炎症性肠病问卷自我填写指导

本问卷是用来测量炎症性肠病对您日常功能和生存质量的影响。它将询问您肠病给您带来的症状，您的总体感觉和心情。

本问卷共有32 个问题。每个问题均设有从1 到7 不同程度的答案。请仔细阅读每个问题，并选择最能反映您过去2 周感受的1个答案。

例如：

过去2 周，您因肠道问题而感到身体不适的频率是：

1 所有时间

2 大部分时间

3 很多时间

4 有些时间

5 少部分时间

6 很少时间

7 完全没有

如果您对某个问题不甚理解，请暂停片刻，思考一下该问题对你的意义，您的肠道问题如何影响到它，然后尽最大努力回答该问题。您只需花您几分钟时间来完成。

炎症性肠病问卷

本问卷是用来调查您最近2周的感受。询问您因炎症性肠病引起的症状，您的总体感觉和心情。

1．过去2 周,您的大便次数有多频繁？请选择下列其中一项以反映过去2 周您的大便频率：

□1 大便次数跟过去最严重时一样频繁或更频繁

□2 极度频繁

□3 非常频繁

□4 大便次数中度增加

□5 大便次数有些增加

□6 大便次数稍有增加

□7 正常，大便次数没有增加

2． 过去2 周，您有多少时间受到疲劳、乏力或筋疲力尽感的影响？请选择下列其中一项以反映过去2 周内您因疲劳、乏力而受影响的时间：

□1 所有时间

□2 大部分时间

□3 很多时间

□4 有些时间

□5 少部分时间

□6 很少时间

□7 完全没有

3． 过去2 周，您有多少时间感到沮丧、不耐烦或烦躁不安？请从下列选项中选择一项：

□1 所有时间

□2 大部分时间

□3 很多时间

□4 有些时间

□5 少部分时间

□6 很少时间

□7 完全没有

4． 过去2 周， 您有多少时间因肠道问题而不能上学或工作？请从下列选项中选择一项：

□1 所有时间

□2 大部分时间

□3 很多时间

□4 有些时间

□5 少部分时间

□6 很少时间

□7 完全没有

5． 过去2 周，您有多少时间有解稀便的现象？请从下列选项中选择一项：

□1 所有时间

□2 大部分时间

□3 很多时间

□4 有些时间

□5 少部分时间

□6 很少时间

□7 完全没有

6． 过去2 周，您有多少精力？请从下列选项中选择一项：

□1 完全没有精力

□2 精力很少

□3 少许精力

□4 有些精力

□5 中等量的精力

□6 精力很多

□7 精力旺盛

7． 过去2 周，您有多少时间担心您的肠道问题可能需要手术治疗？请从下列选项中选择一项：

□1 所有时间

□2 大部分时间

□3 很多时间

□4 有些时间

□5 少部分时间

□6 很少时间

□7 完全没有

8. 过去2 周，您有多少时间因肠道问题而不得不推迟或取消社交活动？请从下列选项中选择一项：

□1 所有时间

□2 大部分时间

□3 很多时间

□4 有些时间

□5 少部分时间

□6 很少时间

□7 完全没有

9. 过去2 周，您有多少时间因腹部绞痛而烦恼？请从下列选项中选择一项：

□1 所有时间

□2 大部分时间

□3 很多时间

□4 有些时间

□5 少部分时间

□6 很少时间

□7 完全没有

10. 过去2 周，您有多少时间感到身体不适？请从下列选项中选择一项：

□1 所有时间

□2 大部分时间

□3 很多时间

□4 有些时间

□5 少部分时间

□6 很少时间

□7 完全没有

11. 过去2 周，您有多少时间因担心找不到厕所而烦恼？请从下列选项中选择一项：

□1 所有时间

□2 大部分时间

□3 很多时间

□4 有些时间

□5 少部分时间

□6 很少时间

□7 完全没有

12． 过去2 周，肠道问题给您原本想参加的休闲或体育活动带来多大困难？请从下列选项中选择一
项：

□1 很大困难，无法进行活动

□2 很多困难

□3 中等度困难

□4 有些困难

□5 很少困难

□6 极少困难

□7 没有困难，肠道问题没有限制体育或休闲活动

13． 过去2 周，您有多少时间因腹痛而烦恼？请从下列选项中选择一项：

□1 所有时间

□2 大部分时间

□3 很多时间

□4 有些时间

□5 少部分时间

□6 很少时间

□7 完全没有

14． 过去2 周，您有多少时间因夜间不能安睡或夜间醒来而烦恼？请从下列选项中选择一项：

□1 所有时间

□2 大部分时间

□3 很多时间

□4 有些时间

□5 少部分时间

□6 很少时间

□7 完全没有

15． 过去2 周，您有多少时间感到抑郁或沮丧？请从下列选项中选择一项：

□1 所有时间

□2 大部分时间

□3 很多时间

□4 有些时间

□5 少部分时间

□6 很少时间

□7 完全没有

16． 过去2周，您有多少时间因您想要去的场所附近没有厕所而去不了？请从下列选项中选

择一项：

□1 所有时间

□2 大部分时间

□3 很多时间

□4 有些时间

□5 少部分时间

□6 很少时间

□7 完全没有

17. 总的说来，过去2 周，大量放屁对您来说是一多大问题？请从下列选项中选择一项：

□1 是一严重问题

□2 是一重大问题

□3 是一明显问题

□4 有些麻烦

□5 很少麻烦

□6 绝少麻烦

□7 没有麻烦

18. 总的说来，过去2 周，保持或达到您想要的理想体重对您来说是一多大问题？请从下列选项中
选择一项：

□1 是一严重问题

□2 是一重大问题

□3 是一明显问题

□4 有些麻烦

□5 很少麻烦

□6 绝少麻烦

□7 没有麻烦

19. 许多肠病病人经常会因疾病而担心、忧虑。包括担心并发癌症、担心病情不会好转，担心复发。
总体来说，过去2 周，您有多少时间感到这方面的担心、忧虑？请从下列选项中选择一项：

□1 所有时间

□2 大部分时间

□3 很多时间

□4 有些时间

□5 少部分时间

□6 很少时间

□7 完全没有

20. 过去2 周，您有多少时间因腹胀而烦恼？请从下列选项中选择一项：

□1 所有时间

□2 大部分时间

□3 很多时间

□4 有些时间

□5 少部分时间

□6 很少时间

□7 完全没有

21． 过去2 周，您有多少时间感到放松、没有压力？请从下列选项中选择一项：

□1 完全没有

□2 少部分时间

□3 有些时间

□4 很多时间

□5 大部分时间

□6 几乎所有时间

□7 所有时间

22． 过去2 周，您有多少时间在排便时有直肠出血的问题？请从下列选项中选择一项：

□1 所有时间

□2 大部分时间

□3 很多时间

□4 有些时间

□5 少部分时间

□6 很少时间

□7 完全没有

23． 过去2 周，您有多少时间因您的肠道问题而感到尴尬？请从下列选项中选择一项：

□1 所有时间

□2 大部分时间

□3 很多时间

□4 有些时间

□5 少部分时间

□6 很少时间

□7 完全没有

24． 尽管肠道是空的，但仍感觉要上厕所，过去2 周，您有多少时间为此而烦恼？请从下列选项中
选择一项：

□1 所有时间

□2 大部分时间

□3 很多时间

□4 有些时间

□5 少部分时间

□6 很少时间

□7 完全没有

25. 过去2 周，您有多少时间感到伤心流泪或心理难过？请从下列选项中选择一项：

□1 所有时间

□2 大部分时间

□3 很多时间

□4 有些时间

□5 少部分时间

□6 很少时间

□7 完全没有

26. 过去2 周，您有多少时间因意外弄脏内裤而烦恼？请从下列选项中选择一项：

□1 所有时间

□2 大部分时间

□3 很多时间

□4 有些时间

□5 少部分时间

□6 很少时间

□7 完全没有

27. 过去2 周，您有多少时间因肠道问题而感到愤怒？请从下列选项中选择一项：

□1 所有时间

□2 大部分时间

□3 很多时间

□4 有些时间

□5 少部分时间

□6 很少时间

□7 完全没有

28. 过去2 周，您的肠道问题在多大程度上限制了您的性生活？请从下列选项中选择一项：

□1 因肠病之故没有性生活

□2 因肠病之故严重受限

□3 因肠病之故中度受限

□4 因肠病之故有一些限制

□5 因肠病之故稍有限制

□6 极少因肠病之故受限制

□7 并未因肠病而受限制

29. 过去2 周， 您有多少时间因恶心、胃部不适而烦恼？请从下列选项中选择一项：

□1 所有时间

□2 大部分时间

□3 很多时间

□4 有些时间

□5 少部分时间

□6 很少时间

□7 完全没有

30． 过去2 周，您有多少时间感到急躁易怒？请从下列选项中选择一项：

□1 所有时间

□2 大部分时间

□3 很多时间

□4 有些时间

□5 少部分时间

□6 很少时间

□7 完全没有

31． 过去2 周，您有多少时间感到缺乏他人的理解？请从下列选项中选择一项：

□1 所有时间

□2 大部分时间

□3 很多时间

□4 有些时间

□5 少部分时间

□6 很少时间

□7 完全没有

32． 过去2 周，您对个人生活感到有多满意、幸福或开心？请从下列选项中选择一项：

□1 大部分时间感到非常不满意、不幸福

□2 总体来说不满意、不幸福

□3 有些不满意、不幸福

□4 总体来说满意、开心

□5 大部分时间感到满意、幸福

□6 大部分时间感到非常满意、幸福

□7 特别满意，没有比现在更幸福、开心了
